# Type II Heat-Labile Enterotoxins from 50 Diverse *Escherichia coli* Isolates Belong Almost Exclusively to the LT-IIc Family and May Be Prophage Encoded

**DOI:** 10.1371/journal.pone.0029898

**Published:** 2012-01-05

**Authors:** Michael G. Jobling, Randall K. Holmes

**Affiliations:** Department of Microbiology, University of Colorado School of Medicine, Aurora, Colorado, United States of America; National Institutes of Health, United States of America

## Abstract

Some enterotoxigenic *Escherichia coli* (ETEC) produce a type II heat-labile enterotoxin (LT-II) that activates adenylate cyclase in susceptible cells but is not neutralized by antisera against cholera toxin or type I heat-labile enterotoxin (LT-I). LT-I variants encoded by plasmids in ETEC from humans and pigs have amino acid sequences that are ≥95% identical. In contrast, LT-II toxins are chromosomally encoded and are much more diverse. Early studies characterized LT-IIa and LT-IIb variants, but a novel LT-IIc was reported recently. Here we characterized the LT-II encoding loci from 48 additional ETEC isolates. Two encoded LT-IIa, none encoded LT-IIb, and 46 encoded highly related variants of LT-IIc. Phylogenetic analysis indicated that the predicted LT-IIc toxins encoded by these loci could be assigned to 6 subgroups. The loci corresponding to individual toxins within each subgroup had DNA sequences that were more than 99% identical. The LT-IIc subgroups appear to have arisen by multiple recombinational events between progenitor loci encoding LT-IIc1- and LT-IIc3-like variants. All loci from representative isolates encoding the LT-IIa, LT-IIb, and each subgroup of LT-IIc enterotoxins are preceded by highly-related genes that are between 80 and 93% identical to predicted phage lysozyme genes. DNA sequences immediately following the B genes differ considerably between toxin subgroups, but all are most closely related to genomic sequences found in predicted prophages. Together these data suggest that the LT-II loci are inserted into lambdoid type prophages that may or may not be infectious. These findings raise the possibility that production of LT-II enterotoxins by ETEC may be determined by phage conversion and may be activated by induction of prophage, in a manner similar to control of production of Shiga-like toxins by converting phages in isolates of enterohemmorhagic *E. coli*.

## Introduction

Enterotoxigenic *Escherichia coli* (ETEC) are the most common cause of bacterial diarrhea amongst travelers and overseas military personnel [1]. They also cause significant mortality and morbidity among infants in the developing world, with estimates of 280–650 million cases reported for children under five and up to 800,000 fatalities a year [2], [3]. ETEC colonize but do not invade the small intestine, where they produce the toxin(s) largely responsible for the profuse watery diarrhea, either a heat-labile enterotoxin (LT) or a heat-stable enterotoxin (ST), or both. LT was first discovered in porcine and calf ETEC isolates in 1967 and in human ETEC isolates in 1971 [4]. The genes encoding LT are found as a plasmid-encoded operon, consisting of translationally coupled A and B genes whose products are secreted to the periplasm, where they assemble into a heterohexameric complex of one A polypeptide and five B polypeptides. LT is highly related to and immunologically cross-reactive with cholera toxin (CT). The toxin binds to cell surface gangliosides on enterocytes via the B pentamer, and delivers the enzymatically active A1 subunit to the cytosol where it ADP-ribosylates and constitutively activates the stimulatory G protein G_s_α. This leads to increased production of cAMP and subsequent chloride and electrolyte secretion into the gut lumen producing a profuse watery diarrhea. In the early 1980's it became clear that some ETEC isolates did not make LT or ST but produced an antigenically unrelated LT-like activity [5]; these were isolated from diverse sources – humans, animals and foodstuffs [6]. The toxins produced by such ETEC isolates were termed type II heat-labile enterotoxins to distinguish them from the CT and LT-I enterotoxin group. The prototype LT-II enterotoxin (LT-IIa) was purified and the operon that encodes it was cloned and sequenced from an ETEC isolate from a water buffalo [7], [8], [9]. A second type II enterotoxin (LT-IIb) and the operon that encodes it in an ETEC isolate from a cooked beef sample was subsequently characterized [10]. LT-IIa and LT-IIb are encoded by highly related operons that are more distantly related to the operons that encode CT and LT-I. While the mature LT-IIa and LT-IIb A polypeptides are 84% identical to each other and 57–59% identical to CT/LT, the mature LT-IIa and LT-IIb B polypeptides are only 57% identical to each other and 15–16% identical to the mature CT/LT-I B subunits. Nevertheless the crystal structures of CT, LT-I and LT-IIb holotoxins reveal that their folds are very similar [11]. All members of this family are excellent adjuvants and have strong immunomodulatory properties that vary between toxins [12] that may aid in vaccine design and development. Isolation and characterization of additional novel variants of enterotoxins may be useful in expanding the repertoire of immunomodulatory enterotoxins.

As ETEC isolates are indistinguishable from normal flora by standard microbiological tests, they are often overlooked. The Centers for Disease Control [13] state that “ETEC should be suspected in outbreaks of gastroenteritis when common bacterial or viral enteric pathogens are not identified”. Sjöling et al. [14] state that “ETEC has, however, been grossly underestimated as the cause of diarrhea in many studies, mainly because few laboratories have suitable methods in place for ETEC diagnostics”. Current diagnostic techniques in use include ELISA and PCR-based assays to detect LT-I/ST ETEC, and due to time and cost issues their use has largely displaced tissue-culture based assays that detect enterotoxins by biological activity. Thus the prevalence of LT-II producing ETEC is largely unknown. A retrospective study by Seriwatana et al [15] found that in a collection of 141 ST- LT+ ETEC isolates collected from humans, animals and foods that still showed enterotoxic activity on Y1 cells, 22% were LT-II+ by colony hybridization (41/141; 6% of these were human isolates).

Southern blots using probes specific for LT-IIa and LT-IIb genes [7], [10] were performed on several of these isolates and indicated that their LT-II loci encoded variants of LT-II that were related to, but distinct from, LT-IIa and LT-IIb. In this study we developed PCR-based methods to isolate and characterize the LT-II loci in 48 additional type II ETEC isolates from humans (20%), animals (60%) and food (20%) sources, and we found that loci encoding LT-IIa and LT-IIb were very uncommon among the isolates in our collection. Most of the predicted LT-II toxins form a single group that we designate the LT-IIc family. One subgroup of this toxin family is identical to the recently described LT-IIc of Nawar et al. [16] who, while this study was in progress, independently cloned and characterized a novel LT-II operon from an ETEC isolated from ostriches that had diarrhea and died suddenly [17]. Here we present an analysis of the toxin-encoding genetic loci and deduced protein sequences from a diverse group of ETEC isolates whose toxins now form the LT-IIc family of enterotoxins.

## Materials and Methods

### Bacterial strains and media

Our collection of type II ETEC isolates are listed in table 1, along with their known properties, isolation dates and sources. This collection, accumulated over several decades from various ETEC researchers based around the globe, from clinical, veterinary and food microbiology laboratories, represents an unbiased selection of uncharacterized type II ETEC strains. These isolates were maintained since receipt as glycerolized cultures at −80°C. For routine cultures, isolates were streaked onto LB plates and grown overnight at 37°C.

**Table 1:** Sources, phenotypes and properties of type II ETEC isolates.

Strain	LT-II type	*E. coli* phylo-type	Isolated from	Diarrhea^a^	Country and Year of isolation^b^		From^c^	Serotype, other details^d^
SA53-1	IIa	B1	buffalo	no	Thailand	1980	PE	O103:H21 EHEChly+ VT2+ [7], [8], [9], [15] e
P393F-10	IIa	A1	human adult	no	Philippines	1979	PE	O109:H21 Philippine waitress
C2 94	IIa	D1	buffalo	yes	Sri Lanka	1986*	PE	VT2+ [15]
T2 41	IIb	B1	cooked beef	food sample	Brazil	1984*	LT	O8:H21 or O75:H21 [10]
357900	IIc1	B1	human adult	yes	Bangladesh	1985	PE	Originally from K. Timmis [53]
T3 IAL190	IIc1	A1	raw beef	food sample	Brazil	1984*	LT	O8:H21
C9 300	IIc1	D1	calf	not known	Sri Lanka	1986*	PE	
230A	IIc1	B1	calf	yes	Belgium	1967	JM	8 day old calf
NADC567	IIc1v	B1	calf	yes	USA	1963	TC	O36:K+:H42
NADC1036	IIc1v	B1	calf	yes	USA	1963	TC	O88:K+;H16
336A	IIc1v	B1	calf	yes	Belgium	1968	JM	2 day old calf
30401-3	IIc1v	D1	calf	yes	Belgium	1982	JM	1 month old calf
30580-3	IIc1v	D1	calf	yes	Belgium	1982	JM	2 day old calf
T4 442/2	IIc2	B1	human child	yes	Brazil	1979	LT	O112:H21
T5 0-4	IIc2	B1	human adult	yes	Brazil	1979	LT	O1:H8
T7	IIc2	B1	sausage	food sample	Brazil	1984*	LT	O141:H16
T8 S38	IIc2	A1	sausage	food sample	Brazil	1984*	LT	O141:H16
T9	IIc2	B1	sausage	food sample	Brazil	1984*	LT	O141:H16
PS23-2	IIc2	B1	human adult	no	Thailand	1980	PE	Am Peace Corp Volunteer O71:H7
PC34-2	IIc2	B1	human adult	no	Thailand	1979	PE	Am Peace Corp Volunteer O115:H28
SVDV23-5	IIc2	B1	human adult	no	Thailand	1980*	PE	O7:H8
C8 259	IIc2	D1	calf	no	Sri Lanka	1986*	PE	VT2+ [15]
NK87-1	IIc2	B1	water buffalo	?	Thailand	1984*	PE	
SPK14BIB-3	IIc2	B1	cow or buffalo	?	Thailand	1984*	PE	
E21485/O/A	IIc2	B1	human adult	yes	Thailand	1984*	SS	O71:H7
NADC2044	IIc2	B1	sheep	dead	USA	1974	TC	O15:H21 CNF2+ F17+ strain S5 [61], [62]
NADC476	IIc2	B1	cow	dead	USA	1963	TC	
33592-1	IIc2	B1	calf	yes	Belgium	1985	JM	Experimental calf infected with rotavirus
35242-1	IIc2	B1	calf	yes	Belgium	1985	JM	3 week old septicemic calf after necropsy
D217-5	IIc3	B1	human child	yes	Thailand	1985	PE	Bangkok 5 yr old [15]
T1 34/4	IIc3	B1	mayonnaise	food sample	Brazil	1984*	LT	O11:H28
C6 210	IIc3	A1	calf	not known	Sri Lanka	1986*	PE	
F150-4	IIc3	A1	beef	food sample	Thailand	1986	PE	Bangkok market [63]
F162-4	IIc3	D1	beef	food sample	Thailand	1986	PE	Bangkok market [63]
WY517-2	IIc4	B1	human child	yes	Africa	1984*	PE	Somalia [15]
SA35-3	IIc4	B1	buffalo	?	Thailand	1980	PE	O6:H-
C4 149	IIc4	D1	calf	not known	Sri Lanka	1986*	PE	
C5 207	IIc4	B1	calf	not known	Sri Lanka	1986*	PE	
C7 215	IIc4	A1	calf	not known	Sri Lanka	1986*	PE	
F250-3	IIc4	A1	beef	food sample	Thailand	1986	PE	Bangkok market [63]
35227-1	IIc4	B1	calf	yes	Belgium	1985	JM	rotavirus infected experimental 3 d old calf
35227-2	IIc4	B1	calf	yes	Belgium	1985	JM	rotavirus infected experimental 3 d old calf
SA31-1	IIc5	B1	buffalo	?	Thailand	1980	PE	O103:H-
C3 120	IIc5	B1	calf	not known	Sri Lanka	1986*	PE	
F214-2	IIc5	B1	beef	food sample	Thailand	1986	PE	Bangkok market [63]
SA100 2nc	IIc5v	B1	buffalo	?	Thailand	1980	PE	
SA76-4	IIc5v	B1	cow	?	Thailand	1980	PE	O117:H21
C1 31	IIc6	B1	calf	not known	Sri Lanka	1986*	PE	C1–C9 originally from S. Peiris
3PS2-2	?	B1	human adult	no	Thailand	1980	PE	Am Peace Corp Volunteer O15:H-

a ? Animal isolates from Thailand via PE were obtained from rectal swabs of animals kept under the house of an index case of diarrhea in rural Thai villagers; these animals had chronically loose stools, but were not recognized as having acute diarrhea. Details are as reported to us by the provider for each isolate. The disease state of animals from which isolates C1, C3–C7 and C9 were recovered was not reported to us and are shown as unknown; C2 and C8 details were reported in [15].

b * approximate - actual date of isolation not available.

c PE, Peter Echeverria; LT, Luis Trabulsi (deceased); SS, Silvia Scotland; TC, Tom Casey; JM, Juan Mainil.

d VT2+, SLT-II probe positive; EHEChly+, EHEC plasmid probe positive; CNF2+, expresses cytotoxic necrotizing factor 2; F17+, F17 fimbriae positive.

e References.

### DNA methods, isolation and PCR conditions

Restriction and DNA modifying enzymes were used according to manufacturer's conditions, and were from Fermentas Life Sciences (now Thermo Fisher) unless otherwise stated. *E. coli* TE1 strain [18] was made competent for transformation using the Z-Competent buffer set (Zymo Research). A modified chromosomal DNA isolation procedure based on that of Chen and Kuo [19] was used. Briefly, a large loopful of cells was scraped from a fresh LB plate and resuspended in 400 µL of TE (10 mM Tris-HCl, pH 8.0, 1 mM EDTA), and lysed with 1/10^th^ volume of 10% SDS. Proteins were precipitated by mixing with 1/3^rd^ volume 5 M NaCl and removed by centrifugation, followed by one phenol:chloroform extraction. DNA was precipitated with 0.6 volume propan-2-ol and spooled out on a tip, transferred to a 70% ethanol wash in a fresh tube, pelleted for 30 seconds in a microfuge, gently dried at 37°C then dissolved in 250 µL TE.

PCR was done in 1× Taq buffer with (NH_4_)_2_SO_4_ and 1.5 mM MgSO_4_ (Fermentas Life Sciences) with either of Pfu or Taq polymerase at 2.5 U/100 µL reaction, 1 µM of each primer and 1 µL of a template dilution. An initial denaturation was done at 94°C for 3 mins, followed by 25–35 cycles of 94°C denaturation for 10 secs, 45–55°C primer annealing for 10 secs and 72°C extensions for 30 (Taq) or 60 (Pfu) secs per kb. Primers were synthesized by Invitrogen or IDT, dissolved in TE at 100 µM and used without further purification.

Inverse PCR was performed by digesting 0.9 µg of chromosomal DNA to completion with *Pst*I in 10 µL; 180 ng (2 µL) of this was then diluted into 90 µL 1× ligase buffer with PEG (Fermentas) and self-ligated with 2 U T4 DNA ligase, for 2 hours at room temperature. One µL of this ligation was then used as template for inverse PCR in 50 µL using SpeedStar polymerase (TaKaRa Bio) with an initial denaturation of 94°C for 4 mins followed by 30 cycles of 94°C for 10 secs, 60°C for 10 secs and 72°C for 60 seconds.

Walking PCR was done essentially as described by Pilhofer et al., [20]. This two-step method first performs a specific 30-cycle linear amplification using one specific outward primer, followed by a single cycle with reduced stringency annealing, when the primer may use the reverse strand of the linear products as template to anneal at sub-optimal sites. Another 30 cycles of exponential PCR follows where the single primer amplifies the less-specifically primed products of the reduced stringency cycle. Subsequently direct sequencing of the reaction products using a nested specific outward primer directly generates sequence for the region 3′ of the known sequence.

Determination of *E. coli* phenotypic group by triplex PCR for *chuA*, *yjaA* and TspE4.C2 (putative lipase) was done essentially as described [21] using the simplified two-step reaction, except with 0.2 µM primers in a 12 µL reaction volume with 1 µL of boiled cell lysate or chromosomal DNA, using DreamTaqGreen polymerase (Fermentas Life Sciences) at 1.5 U per 100 µL.

### Sequence analysis

Dye terminator DNA sequencing was done by the CU Cancer Center core facility at the University of Colorado Anschutz Medical Campus. Sequence assembly and analysis was done with the Clone Manager Professional software suite (SciEd software) using ClustalW with default parameters (mismatch -2, open gap 4 and extend gap 1 penalties). Phylogenetic analysis was done with MEGA5 software [22]. Sequence data presented for representative LT-IIc loci have been deposited in Genbank with the accession numbers JQ031705 - JQ031710 for LT-IIc1 through LT-IIc6 and the extended sequences for LT-IIa and LT-IIb loci assigned JQ031711 and JQ031712 respectively.

## Results

### Amplification of the type II enterotoxin A1 gene of strain 442/2 by degenerate PCR

**Figure 1:**
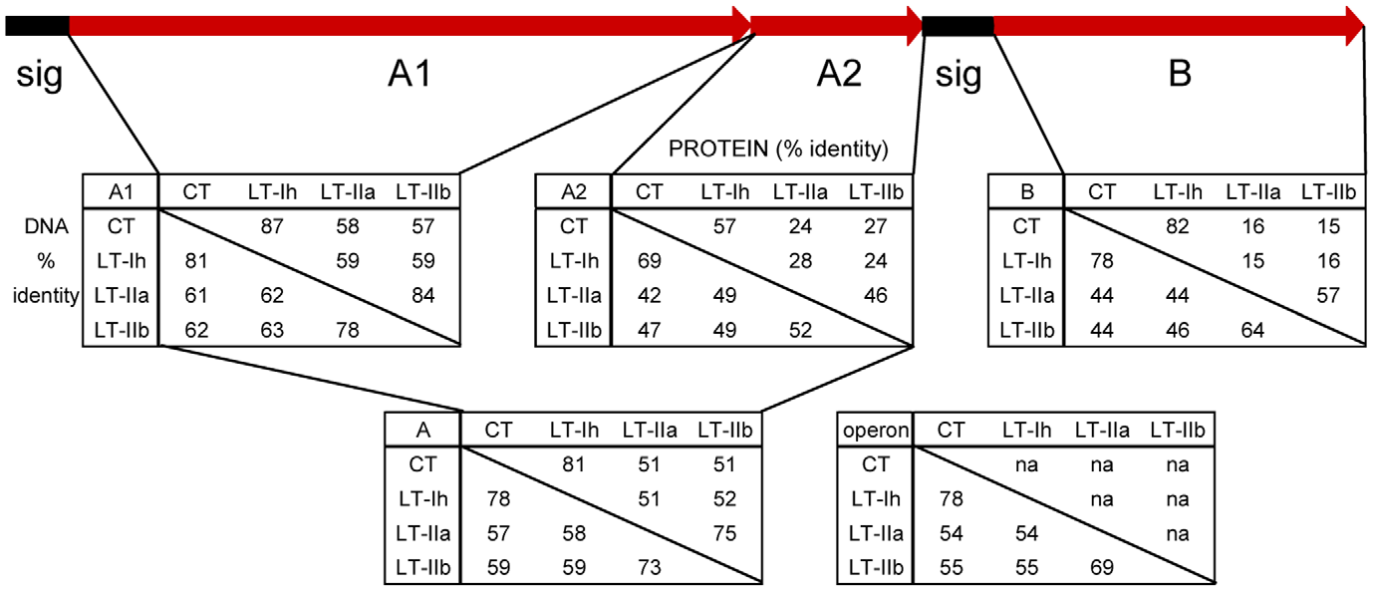
Heat-labile enterotoxin gene organization showing DNA sequence and protein identity between operons, polypeptides and subunits. Genes are shown as red arrows (A1, enzymatic domain, A2, linker domain between A1 and B pentamer, B, binding subunit monomer) with signal sequences (sig) shown as black boxes. Below the genes boxes show percent identity for DNA (lower half) or protein (Upper half) for the corresponding regions. Signal sequences are only included in percent identity comparisons for the complete operon.

Figure 1 shows the operon structure of a typical enterotoxin locus, and the percent identities at the nucleotide level for the complete operons for the cholera toxin, LT-I, LT-IIa and LT-IIb loci, and the nucleotide and amino-acid identities for the toxin subdomains (A, A1, A2 and B). Percent identities are highest for the A1 domains (at the mature polypeptide level, 87% for CT versus LT-I, and 58 or 57% for CT versus LT-IIa or LT-IIb), reduced for the A2 domains (for CT-A2 polypeptide only 24% versus LT-IIa-A2 up to 57% versus LT-I-A2), and lowest for the B genes (only 15–16% identity for CT or LT-I versus LT-IIa or LT-IIb B subunits). The percentages are higher when comparing only the LT-IIa and LT-IIb toxins, with the mature A1 coding sequences being 84% identical, and the LT-IIa and LT-IIb B subunits showing 57% identity. From these comparisons we identified two regions near the termini of the A1 subunits that were sufficiently conserved to permit design of PCR primers for attempted amplification of most of the A1 coding sequence from ETEC isolates with uncharacterized enterotoxin loci.

The highly conserved amino-acid sequence RADSR(T/P)PDE is centered around the catalytic arg-7 residue of the 192 residue mature cholera toxin A1 subunit, while the somewhat less conserved sequence near the carboxyl-terminus of the A1 subunit, AW(R/E)E(E/M/V)PW, ends at trp-179 (Figure S1). Additionally, primers based on these sequences could be designed with restriction sites enabling cloning of the PCR product back into a holotoxin operon, by introducing an *Xba*I site (TCTAGA, naturally present in *ctxA*) at the conserved SR peptide and an *Nco*I site (CCATGG, naturally present in the LT-IIa-A gene) at the conserved PW peptide.

We designed forward and reverse degenerate DNA primers encoding these residues, LTXba5 (GAGCwGAy*TCTAGA*mCnCCwGAyGA) and LTNco3 (Gy *CCATGG* CwyTTCyyyCCAnGC) (constrained restriction sites in italics) which have 32-fold and 256-fold degeneracy respectively with only 4-fold degeneracy within the six nucleotides at the 3′ ends.

Degenerate PCR was done using an annealing temperature of 45°C, and products were initially obtained from two isolates, 442/2 (T4) and P393-F10 that were isolated from humans, one with and one without diarrhea respectively. *Xba*I and *Nco*I digested PCR products were cloned into pMGJ148, a cholera holotoxin expression vector derived from pMGJ142 [23] with an added *Nco*I site at the Pro-Trp codons in *ctxA*, followed by DNA sequencing of the cloned novel A1 encoding genes.

**Figure 2:**
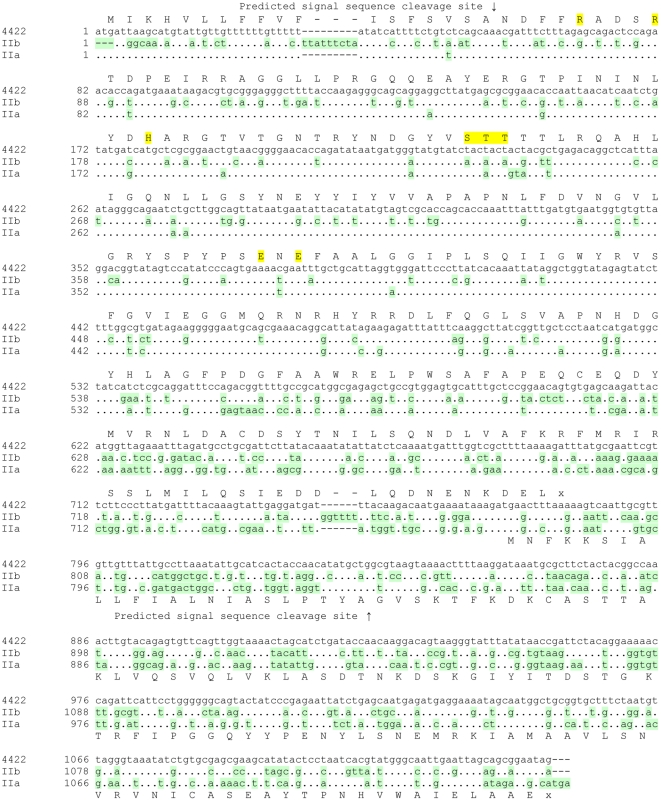
Nucleotide comparison of the full LT-II operon from 442/2 against LT-IIa and LT-IIb operons. Translations for the 442/2 A gene are shown above the sequence and for the B gene below the sequence. Identities are shown as periods, differences are shown shaded light green and enzymatically important residues are highlighted in yellow. Positions of predicted signal sequence cleavage sites are indicated by up or down arrows respectively for the A and B polypeptides.

The DNA sequence obtained for the PCR-amplified unique region of the *Xba*I-*Nco*I DNA fragment from the P393-F10 isolate was identical to that for LT-IIa, while that from the 442/2 isolate was novel (Figure 2). At the nucleotide level the 442/2 sequence showed 91% and 80% identity to LT-IIa and LT-IIb respectively, while the encoded amino-acids were 91% and 87% identical respectively (Figure S2). The predicted A1 protein from 442/2 differed at 8 positions from both the LT-IIa and LT-IIb A1 polypeptides, and it differed at 6 additional residues from LT-IIa only and at 13 additional residues from LT-IIb only. Thus the novel portion of the A1 subunit was more similar to LT-IIa than to LT-IIb. Furthermore, 10 of the 14 differences between the 442/2 and LT-IIa A1 polypeptides occurred in the last 40 amino-acids, and the 442/2 A1 polypeptide differed from the LT-IIa A1 polypeptide at only 3 of the first 118 residues (97.5% identity).

### Walking PCR to obtain the A2 and B gene sequences

We used the walking PCR method of Pilhofer et al., [20] to generate DNA sequence information for the genes immediately 3′ of the novel LT-II A1 gene in the 442/2 locus. From the product of this reaction using 442/2 DNA as template we obtained approximately 540 bp of readable sequence, just sufficient to identify the A2 gene and the complete B gene. Given the high sequence identity between the A1 polypeptides of 442/2 and LT-IIa, it was surprising to see that they shared only 37% amino-acid identity in their A2 subunits (Figure 2); indeed the identity was marginally higher to LT-IIb at 40%. This pattern carried over into the B gene, where the DNA sequence identity was only 46% to LT-IIa and 58% to LT-IIb, but 53% and 51% at the amino-acid level respectively. Since the LT-II locus of 442/2 differed significantly from both LT-IIa and LT-IIb, we tentatively named this toxin LT-IIc. The DNA sequence presented in Figure 2 includes the amino-terminal portion of the A gene obtained as described in the next section.

### Inverse PCR to obtain the complete 442/2 LTII operon

The DNA sequences obtained by degenerate PCR and PCR-walking covered 93% of the A and B genes but was missing the leader sequence and start of the A gene, and all sequence 3′ and 5′ of the operon. Since the DNA sequence of the predicted 442/2 LTII operon indicated that this locus was novel, we designed 442/2 operon-specific outward facing primers and used inverse PCR to obtain the remainder of the operon and additional 3′ and 5′ flanking sequences. From a library of self-ligated *Pst*I-cut chromosomal DNA from strain 442/2 we obtained a faint 4 kb product using outward primers 4422R2 and 4422NF which were designed based on the A1 gene sequence; nested PCR using iixBF and 44225XR primers gave a specific 3.5 kb internal amplimer, and sequencing the ends of this product using the same primers completed the sequence of the LTII operon and gave some flanking DNA sequence (Figure S3, which includes the locations and sequences of the primers).

Dr. Terry Connell at the University of Buffalo kindly agreed to supply us prior to publication the coding sequence for his novel LT-II locus obtained from an ETEC isolate (OS1) from an ostrich with diarrhea [16]; this locus has now been designated as a novel member of the LT-II family, LTIIc(OS1), and is the prototype for the LT-IIc group. The LT-IIc(OS1) and the 442/2 loci exhibit much greater identity with each other than with the LT-IIa and LT-IIb loci.

Comparison of the 442/2 LT-II operon with the available LT-IIc(OS1) sequences (start codon A gene to stop codon B gene, no flanking sequence data is available, Figure S4) shows 93% identity at the DNA level, 96% identity for the A gene (98% for the A protein, Figure S5) falling to only 86% identity for the B gene (89% for the B protein). The predicted proteins differ at only 6/260 positions within the A polypeptide, while the B polypeptides differ at 12 of 122 positions (Figure S5). Both proteins have predicted signal sequences of 19 and 23 residues for the A and B polypeptides, respectively, which are identical between LT-IIc(OS1) and LT-IIc(442/2). Therefore the mature A and B polypeptides of LT-IIc(OS1) and LT-IIc(442/2) differ at 6 of 240 residues (97.5% identity) and 11 of 99 residues (89% identity), respectively. Thus, the LT-II locus from ETEC strain 442/2 represents a second distinct subgroup of what we now call the LT-IIc family of heat-labile enterotoxins.

### Screening our LT-II strain collection for LT-IIc-type loci

**Figure 3:**
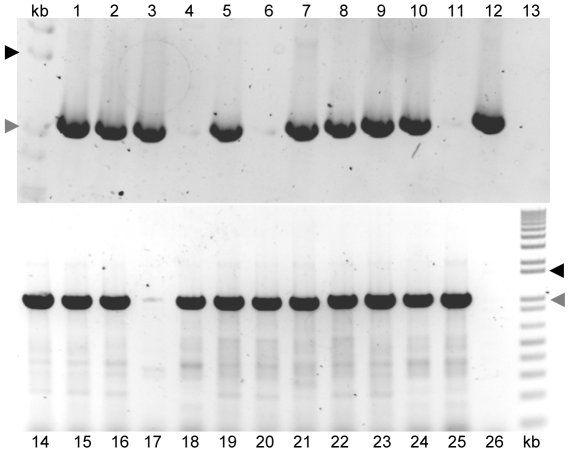
LT-IIc-specific PCR products from 24 ETEC strains in our collection. 1, SA35; 2, SA76; 3, PS23; 4, 3PS2; 5, PC34; 6, P393; 7, SVDV; 8, T1; 9, T3; 10, T5; 11, T6 (LT-I control); 12, T7; 13, no template. 14, T8; 15, T9; 16, C1; 17, C2;18,C3; 19, C4; 20, C5; 21,C6; 22, C7; 23, C8; 24, C9; 25, 357900; 26, no template. kb, kilobase plus ladder (Fermentas Life Sciences). Triangles show marker sizes – black – 1500 bp gray – 1000 bp. Tris-acetate EDTA buffered 0.9% agarose gels run at 200 V for 20 mins (upper panel) or12 mins (lower panel).

Southern blots published by Pickett et al. [7], [10] showed that each of nine other isolates tested from our collection hybridized only weakly with LT-IIa or LT-IIb specific DNA probes, showing that they encoded related but divergent loci. Our novel sequence allowed us to design primers predicted to amplify the A and B genes from both variants of the LT-IIc family. Since the first 100 bp of the A1 gene fragment from 442/2 are 97 and 98% identical to LT-IIa and LT-IIc respectively, a lab stock primer designed to add an *Nde*I site at the start of the mature A1 coding region of LT-IIa was chosen as the forward primer (IIaNdeF), and a reverse primer (IIxBecoR) encoding WAIELA, the carboxyl-terminal end of the B gene lacking the last one (P, 442/2) or two (AE, LTIIc) codons followed by an *Eco*RI site (encoding asn-ser, to enable cloning in-frame to a his-6 tag) was designed de novo (Figure S3). These primers were used to screen 24 isolates randomly selected from our lab collection of type II ETEC isolates (Figure 3). Twenty of the 24 isolates gave a specific single amplicon of the expected size (1.1 kb), indicating that they all belong to the LT-IIc family. One isolate (T6) was a type I ETEC (producing LT-I and ST, data not shown) and it gave no product. Three other isolates, P393-F10, 3PS2-2 and C2 gave no products with these primers. Two of these isolates, P393F-10 and C2, amplified with LT-IIa-specific primers. No other isolate was found that amplified with LT-IIb specific primers. The single isolate 3PS2-2 that gave no product with any primer set may have lost the toxin genes or may encode a novel locus. We have not tested these possibilities. We subsequently tested the other 24 isolates in our collection and found that they also produced products with LT-IIc-specific primers (data not shown).

### The LT-IIc family operons constitute 6 distinct but highly related subgroups

**Figure 4:**
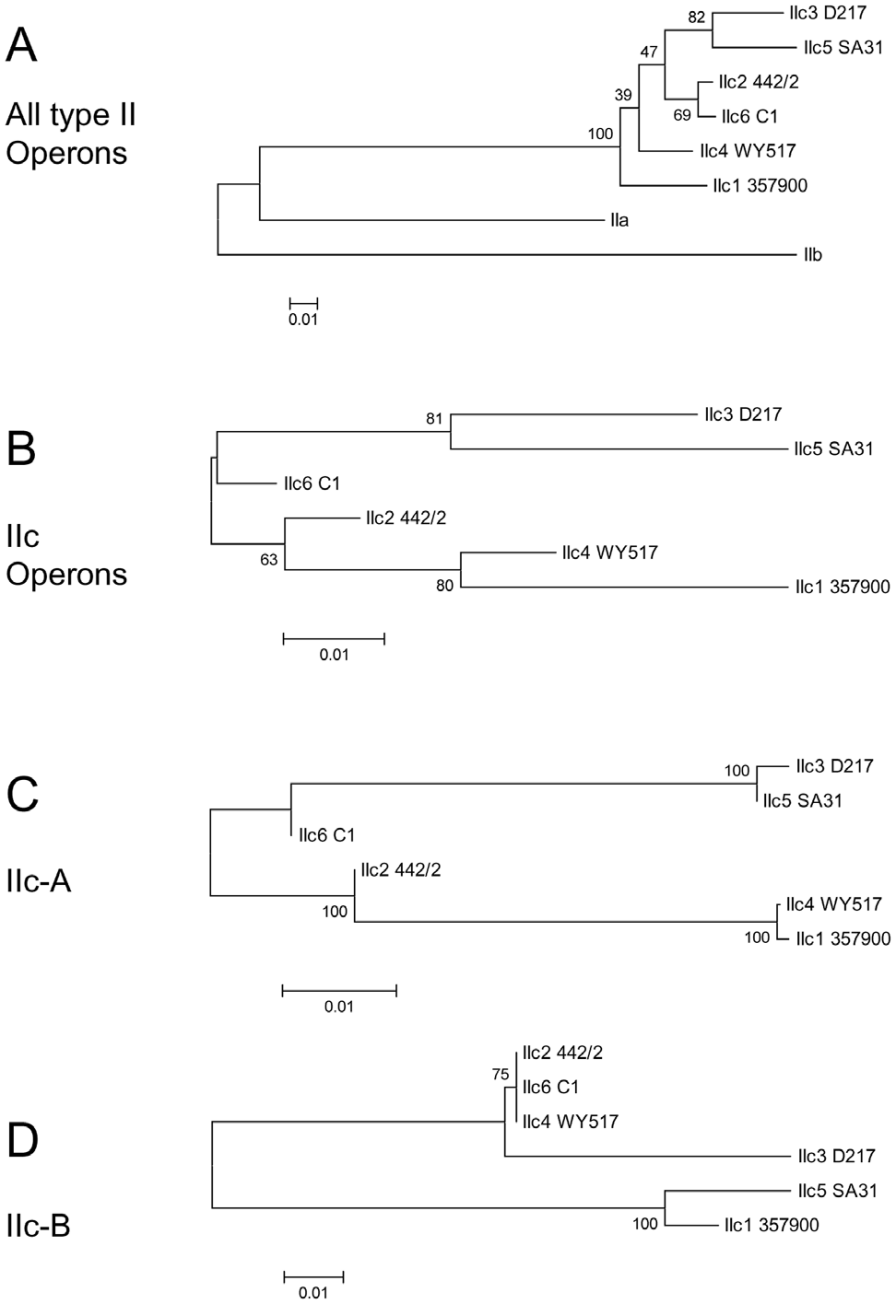
DNA-distance based neighbor-joining phylogeny dendrograms for LT-II loci. The evolutionary history was inferred using the Neighbor-Joining method [58]. The bootstrap consensus tree inferred from 500 replicates is taken to represent the evolutionary history of the taxa analyzed [59]. Branches corresponding to partitions reproduced in less than 50% bootstrap replicates are collapsed. The percentage of replicate trees in which the associated taxa clustered together in the bootstrap test (500 replicates) are shown next to the branches [59]. The trees are drawn to scale, with branch lengths in the same units as those of the evolutionary distances used to infer the phylogenetic tree. The evolutionary distances were computed using the Maximum Composite Likelihood method [60] and are in the units of the number of base substitutions per site. The analyses all involved 6 nucleotide sequences. Codon positions included were 1st+2nd+3rd. All positions containing gaps and missing data were eliminated. There were a total of 1117 (A), 1135 (B,) 780 (C) and 366 (D) positions in the final datasets respectively. Evolutionary analyses were conducted in MEGA5 [22]. A - Dendrogram obtained using all LT-II loci as input sequences B - Dendrogram obtained using only LT-IIc sequences C – Dendrogram obtained using only LT-IIc-A gene sequences D – Dendrogram obtained using only LT-IIc-B gene sequences.

Table 1 lists 50 type II ETEC isolates in our collection and describes their known properties. It includes the type strains for LT-IIa (SA53) and LT-IIb (T2-41). The DNA sequences from the P393-F10 and C2 isolate amplimers with LT-IIa primers were 100% identical to the LT-IIa locus. Forty-six of the remaining isolates gave specific products with the LT-IIc primers. DNA sequences were obtained from the PCR products with the LT-IIc specific primers that covered approximately 90% of the entire operon. Multi-way comparisons including LT-IIa and LT-IIb operons revealed that each of these novel LT-II loci could be assigned to one of six subgroups, typified by isolates 442/2, 357900, D217, WY517, SA31 and C1-31, that these subgroups were clearly separated from LT-IIa and LT-IIb loci and that were closely related to the LT-IIc(OS1) locus from the ostrich ETEC. These operons show a minimum of 88% identity to LT-IIc(OS1) whereas LT-IIa and LT-IIb show only 75% and 67% identity respectively. The DNA-distance based neighbor-joining phylogeny dendrogram tree obtained with MEGA5 software (Figure 4A) shows that all new LT-II operon sequences are rooted together with LT-IIc(OS1 – the DNA sequence is identical to 357900). LT-IIa and LT-IIb branch off from the LT-IIc group very early with LT-IIa being slightly more related to LT-IIc than is LT-IIb. A bootstrap value of 100 (Figure 4A) for the separation of the novel LT-II group from LT-IIa and LT-IIb provides provide strong support for assigning these loci to the novel LT-IIc family. We now subdivide the LT-IIc family into six subgroups, with individual isolates listed in LT-II subgroup order in Table 1. The LT-IIc1 subgroup includes OS1, 375900 and 8 other isolates; the LT-IIc2 subgroup includes 442/2 and 15 other isolates; the LT-IIc3 subgroup includes D217 and 4 other isolates; the LT-IIc4 subgroup includes WY517 and 9 other isolates; the LT-IIc5 subgroup includes SA31 and 4 other isolates, and C1-31 is currently the sole member of the LT-IIc6 subgroup. Within a subgroup the members are more than 99% identical at the nucleotide level over the entire available A and B gene sequences. Table 2 summarizes the properties of the isolates organized by toxin subgroup.

**Table 2:** Summary of ETEC isolate distribution (source, country and phylotype) within each LT-II type.

Toxin subtype	IIa	IIb	IIc1	IIc1v	IIc2	IIc3	IIc4	IIc5	IIc5v	IIc6	Row total
Human diarrhea	-	-	1	-	3	1	1	-	-	-	6
Human no diarrhea	1	-	-	-	3	-	-	-	-	-	4
Animal diarrhea	1	-	1	5	2	-	4	-	-	-	13
Animal unknown	-	-	1	-	2	1	4	2	2	1	13
Animal no diarrhea	1	-	-	-	3	-	-	-	-	-	4
Food	-	1	1	-	3	3	1	1	-	-	10
**Subtotal**	**3**	**1**	**4**	**5**	**16**	**5**	**10**	**3**	**2**	**1**	**50**
Africa	-	-	-	-	-	-	1	-	-	-	1
Bangladesh	-	-	1	-	-	-	-	-	-	-	1
Belgium	-	-	1	3	2	-	3	-	-	-	9
Brazil	-	1	1	-	5	1	-	-	-	-	8
Philippines	1	-	-	-	-	-	-	-	-	-	1
Sri Lanka	1	-	1	-	1	3	3	1	-	1	9
Thailand	1	-	-	-	6	3	2	2	2	-	15
US	-	-	-	2	2	-	1	-	-	-	5
**Subtotal**	**3**	**1**	**4**	**5**	**16**	**5**	**10**	**3**	**2**	**1**	**50**
Phylotype A1	1	-	1	-	1	2	2	-	-	-	7
Phylotype B1	1	1	2	3	14	2	7	3	2	1	37
Phylotype D1	1	-	1	2	1	1	1	-	-	-	6

Within the LT-IIc family as a whole there is evidence of significant genetic rearrangement as some members have highly related A genes but more dissimilar B genes and *vice versa*. Comparing the phylogeny dendrogram trees for these isolates based only on the A or B gene sequences produces two very different trees as compared to the operon-based tree (Figures 4A and B versus 4C, and 4D). The operon tree has notably lower bootstrap values for the nodes than do the A and B gene trees. Comparing B subunit coding sequences, SA31 and D217 are on widely separated arms and yet are closely grouped by A subunit comparisons that have 100% bootstrap values. Similarly, WY517 clusters with 357900/OS1 based on its A gene sequence, but it clusters with 442/2 based on its B gene sequence. The A gene dendrograms have uniform 100% bootstrap confidence levels, indicating that they are correctly grouped, while the operon-based trees have much lower bootstrap values indicating that the confidence in this being the correct tree structure for the operons is less strong. Recombination amongst divergent alleles is the probable reason for obscuring the phylogeny of the complete loci. A close manual analysis of the DNA sequences aligned by ClustalW ([24], Figure S6) reveals probable recombination points and identifies two putatively ancestral LT-II loci in isolates 357900 (LT-IIc1) and D217 (LT-IIc3) from which the other LT-IIc subgroups may be derived by recombination (see discussion). This analysis was largely confirmed using the recombination detection program RDP3 (data not shown) [25].

### Analysis of the LT-IIc A and B polypeptides

The LT-IIc operons encode 259 residue predicted precursor A polypeptides with a minimum 94% identity within the LT-IIc group – decreasing to 79% identity versus LT-IIa A and 70% identity with LT-IIb A, and 121- or 122-residue precursor B polypeptides with a minimum of 87% identity within the LT-IIc group. Both A and B polypeptides have canonical signal sequences of 18 residues for LT-IIc-A and 23 residues for LT-IIc-B polypeptides, giving predicted mature proteins of 241 residues for the A subunit and 98 or 99 residues for the B subunit. All mature A polypeptides have a cysteine pair at 185/197 that is conserved with CT/LT-I/LT-IIa/LT-IIb and likely forms a disulfide loop. Within this eleven residue loop the CT/LT-I/LT-IIa/LT-IIb heat-labile enterotoxin family A polypeptides have a trypsin-cleavable arginine or lysine at residue five. All LT-IIc A polypeptides, however, have a trypsin-cleavable arginine residue at position 7. Cleavage after that arginine residue in mature LT-IIc polypeptides would form a 192-residue A1 subunit and a 49-residue A2 subunit.

The enzymatically active A1 subunits are much more highly conserved within the LT-II family (LT-IIc 442/2 versus other LT-IIc group 96–98% identity; versus LT-IIa, 88% identity and 82% identity versus LT-IIb A1) than are the A2 subunits (85–97% identity within the LT-IIc family, but only 33 and 41% identity between LT-IIc-A2 and LT-IIa-A2 and LT-IIb-A2 respectively). All A polypeptides have conserved catalytically important residues (arg-5, arg-9, ser-thr-ser 59–61 [26], [27], his-42 [28], glu-108 and glu-110 [29] (highlighted in Figure 2).

As is seen for all other members of the heat-labile enterotoxin family, including cholera toxin, the A and B genes for all members of the LT-IIc family overlap by 8 bases (Figure 2) with the B gene ATG codon being within the A gene sequence; This suggest that as for all other members, the genes are translationally coupled,. All predicted mature B polypeptides have either 98 or 99 residues – IIc1 and IIc5 B subunits terminate with ala-97-pro-98 whereas all other IIc B subunits end with ala-97-ala-98-glu-99. Within the LT-IIc family the B subunit amino-acid identities range from 100% down to 86%, but show only 50–54% identity with LT-IIa-B and LT-IIb-B respectively (Figure S5). All LT-IIc B subunits have a conserved cysteine pair that form a disulfide bridge within the monomer, and conserved threonine residues at 13 and 14 that in LT-IIa and LT-IIb B subunits were shown to be important in binding to host cell ganglioside receptors. They all have conservative T34S substitutions with respect to LT-IIa and LT-IIb B subunits at a third residue important for receptor binding. T34S variants of LT-IIa and LT-IIb B subunits retain ganglioside binding activity [30]. It is certainly possible that these variant IIc B subunits are sufficiently divergent as to have distinct ganglioside-binding or immunomodulatory properties, as has already been documented for LT-IIc(OS1) [31]. Confirmation of this possibility will require additional studies.

### Phylogenetic grouping of type II ETEC isolates

*E. coli* isolates can be separated into distinct phylo-groups or ‘subspecies’ that vary in their ecological niches, genome sizes, virulence traits and other biochemical properties [32]. There are four well recognized phylogroups designated A, B1, B2 and D, defined by multilocus enzyme electrophoresis [33] and multi-locus sequence typing [34]. Phylogrouping is thus a valuable tool in initial characterization of *E. coli* isolates. A simple triplex PCR method was developed [21] and validated [35] which correctly assigns 80–85% of all strains to their phylogroup and has 95% accuracy for assignment of group B strains. Table 1 also shows the *E. coli* phylogenetic group (A, B or D) determined for each strain by the triplex PCR method using three loci – *chuA*, *yjaA* and TspE4.C2. Strains are classified based on the presence (+) or absence (−) of these three products (− − − indicates that PCR reactions were negative for all three products and + + + indicates reactions were positive for all three). Group B were the most prevalent at 72% (36 strains) followed by 14% each for A and D groups (seven strains each). Strains negative for all three loci are classified as A0 (− − −, not found), single positives are A1 (− + −, seven strains), B1 (− − +, 36 strains) or D1 (+ − −, seven strains), and double and triple positives are B2_2_ (+ + −, not found), B2_3_ (+ + +, not found), or D2 (+ − +, not found).

### Comparisons of the 3′ and 5′ LT-II operon flanking sequences and encoded polypeptides

When we analyzed the sequence from the inverse PCR product for the flanking DNA sequence on either side of the 442/2 LT-II operon (621 bp 5′ and 1000 bp 3′), we found an open reading frame separated by a 75 bp intervening sequence 5′ of the start codon of the A gene that is predicted to encode a 181-residue protein with 91 and 87% identity to two hypothetical *E. coli* proteins, ERJG_04114 (164/181 residues, E value 7e^−121^) and ECCG_01861, (155/179 residues, E value 6e^−114^)), and 83% identical to a predicted phage lysozyme from *Citrobacter rodentium* ICC168, ROD_36701 (151/181 residues, E value 1e^−112^). In the 1000 bp 3′ of the 442/2 LT-IIc2 B gene we could not identify any open reading frames larger than 50 codons, and we found only a few significant BLAST hits; the most significant match started 460 bp 3′ of the B gene, with 486 bp showing 87% identity to a region (1306591–1307090) of the ETEC strain H10407 genome within a predicted prophage (433/505 nucleotides, E value 1e^−166^); this region has a 50 residue hypothetical putative transmembrane protein whose ORF starts with a GTG codon. Other less significant matches also occur to database entries where surrounding gene products are annotated as potential prophage proteins (data not shown).

**Figure 5:**
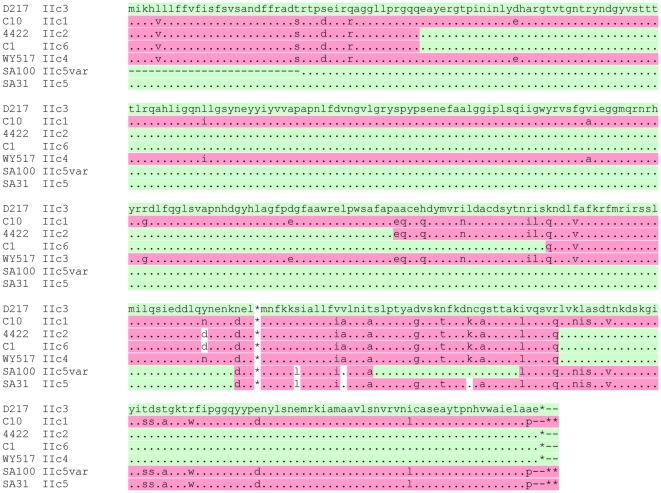
Amino-acid comparison of concatenated A and B polypeptides of representative members of each LT-IIc group. Each protein sequence is compared against LT-IIc3 with identities represented by a period. Regions most similar to LT-IIc3 are colored light green, and those most similar to LT-IIc1 are colored pink. Unshaded residues are presumed substitution variants; * separates the A and B polypeptide sequences.

To further extend the comparison of loci representing the LT-IIc subgroups, we determined the flanking sequences for one member from each of the remaining subgroups (357900 for subgroup IIc1, D217 for subgroup IIc3, WY517 for subgroup IIc4, SA31 for subgroup IIc5 and C1-93 for subgroup IIc6), and also determined flanking DNA sequences for the LT-IIa and LT-IIb loci by extending the DNA sequences of the loci in the published clones (pCP3727 [9] for LT-IIa, 635 bp 5′ and 327 bp 3′; pCP4185 [10] for LT-IIb, 698 bp 5′ and 529 bp 3′) and compared these to the 442/2 locus. A graphical comparison of each locus is shown in Figure 5.

Every LTII locus has a highly conserved ORF, or portion thereof, that is located 5′ of the A gene and is predicted to encode a phage lysozyme. Percent identity for these ORFs to the 442/2 ORF range from 81% for the ORF from LT-IIb up to 93% for the ORF from 357900. In each LT-II locus, this ORF is separated from the LT-II A gene by between 75–89 bp in all but LT-IIb where 246 bp separated the lysozyme and A genes; this intervening sequence is highly conserved, except in the LT-IIb locus. Sequences immediately 3′ of the LTII B gene vary considerably between subgroups, although a unifying feature is the presence of predicted phage-related sequences. The 1000 bp 3′ of the B gene in the LT-IIc4 isolate WY517 is almost identical (98%) to that of LT-IIc2 442/2, as it is for the LT-IIc3 isolate D217 which is 98% identical for the first 826 bp but is then divergent; none of these sequences encode substantial open reading frames. Our LT-IIc1 isolate 357900 locus appears to have a very similar gene organization to that reported for the prototype LT-IIc1 (OS1) isolate [16], with the start for a prophage-encoded *parB*-like gene (best match to *E. coli* K011 and W laboratory K12 strains) located 341 bp downstream of the 357900 LT-IIc1 B gene. The published report for OS1 gives only a graphical representation of the locus with annotations for a lysozyme gene 5′ and a *parB*-like gene 3′ of the LTIIc operon, and does not provide any sequence data. We only have 400 bp of sequence data for the LT-IIc5 3′ sequences for isolate SA31, but these are 99% identical to the 357900 locus and also show the start of a *parB*-like gene. The 3′ sequences for the LT-IIa and LT-IIb loci are unrelated to any of the LT-IIc group or to each other, although both appear to encode the starts of unrelated genes whose best BLAST matches are to putative phage terminase small subunits -ERBG_02499 for the LT-IIa locus (43/50 residues, E value 1e^−10^) and EFF_07592 (39/56 residues, E value 5e^−20^) for the LT-IIb locus.

## Discussion

Until very recently, only two type II ETEC isolates have been well characterized by purification of the protein, with cloning of the operon and determination of the gene sequences [7], [9], [10]. They are the type strains for LT-IIa and LT-IIb toxins respectively. The type II enterotoxin genes from a third strain have recently been cloned and designated LT-IIc [31]. All are chromosomally located. Each isolate that has been characterized has been found to encode related but clearly different LT-II toxin variants. Hence the potential variability within this family is vast. More than 100 instances of ETEC isolates with type II enterotoxin activity or LT-IIa-based DNA probe/PCR signals have been described in the literature with no further characterization as to the nature of the toxin or locus (see later). The frequency of occurrence and distribution of type II ETEC isolates is therefore poorly defined. Additional tools to rapidly and easily determine the presence of type II ETEC isolates are limited and unavailable to most laboratories. Determining the range of variability with the type II family and the genetic context of these loci in each isolate will facilitate development of such tools.

In this study we have characterized the type II enterotoxin loci from an additional 48 ETEC isolates in our collection that were isolated from both symptomatic and asymptomatic humans and animals, and from foodstuffs. Surprisingly we found that the vast majority (46 or 92%) of the isolates form a single subgroup to which the LT-IIc from strain OS1 also belongs, while only two previously uncharacterized isolates encoded LT-IIa, and none encoded LT-IIb. This suggests that LT-II types other than LT-IIc may be uncommon or restricted in prevalence to geographical regions or biological sources that have not yet been sampled. The initial characterization of LT-IIa from SA53 and subsequently LT-IIb from strain 41 was highly fortuitous, since 15 of the 18 type II ETEC isolates in our collection at the time LT-IIa and LT-IIb were characterized are here shown to encode LT-IIc variants. There is only a 0.6% probability of randomly picking one LT-IIa isolate and one LT-IIb isolate from these 18 isolates.

Constraints on protein sequence to retain enzymatic activity provide the most likely explanation for the greater similarity among the A1 polypeptides than among the A2 or B polypeptides in members of the heat-labile enterotoxin family. The A2 polypeptides still have some constraints on their structure that enable them to interact with their cognate B polypeptides to form holotoxins. The B polypeptides, which pentamerize and comprise the receptor-binding domain, are the most divergent polypeptide components of the holotoxins. Such conservation of polypeptide function also limits variability within the encoding DNA sequence and enabled us to design degenerate primers that could amplify the more conserved region encoding the enzymatic domain of the A1 polypeptide from the previously uncharacterized type II ETEC isolate 442/2, isolated from a child with diarrhea in Brazil. The encoded A1 polypeptide from this isolate was quite similar to those of LT-IIa and LT-IIb, showing 91 and 87% identity, respectively. The slightly greater variability at the nucleotide level allowed us to design specific primers for amplification of the flanking sequences, first by walking PCR to determine the A2 and B gene encoding sequences and subsequently by inverse-PCR to determine the complete DNA sequence of the operon. The 442/2 LT-II operon is divergent from those encoding LT-IIa, LT-IIb or OS1 LT-IIc, although it is much more closely related to LT-IIc(OS1). The highly conserved amino-acid sequence of the amino-termini of the mature A polypeptides and the carboxyl end of the B polypeptides of these two LT-IIc variants enabled us to design primers that would prime on both loci. Use of these LT-IIc-specific primers enabled us to amplify toxin loci from 45 of the 47 other LT-II-producing isolates in our collection. Determination of the DNA sequences of the unique portions of these PCR products allowed us to identify and characterize 90% of the coding regions of each locus.

The 46 known LT-IIc loci can be assigned to six subgroups which we have designated LT-IIc1 through LT-IIc6, where members of each subgroup differ at the DNA level by less than 0.5%. The LT-IIc1 subgroup can be subdivided based on DNA sequence alone, one set of five isolates (designated IIc1v in table 1; NADC567, NADC1036, 336A, 30401-3 and 305830-3) having 5 silent wobble bases in codons for residues in the A2 and B genes in a 234 bp region (717 to 940) when compared to the OS1 DNA sequence and our isolates 357900, T3, C9 and 230A (Figure S5). The last three of these same wobble base changes are also present in the IIc5 subgroup, which is highly suggestive of recombination between these alleles.

**Figure 6:**
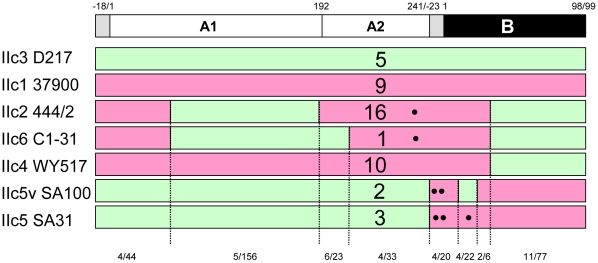
Graphic representation of domain relatedness amongst all LT-IIc groups. Uppermost graphic shows the polypeptide subunits (white or black boxes) with signal sequences as grey boxes. Numbering above these refers to amino-acid positions in the mature protein (negative numbers refer to the signal sequences). Below this graphic are boxes depicting each of the LT-IIc subgroups, LT-IIc1-LT-IIc6, colored as for Figure 4 (green most similar to LT-IIc3, pink most similar to LT-IIc1). Numbers of isolates in each subgroup is indicated within the bar. Black dots denote the approximate positions of individual residue differences within some subgroups (N234D in A, S-18L, A-11V and K9N in B). Numbers at the bottom denote the numbers of residues that differ and the total number of residues in each block.

Recombinational assortment is also seen within the B genes of the LT-IIc family. The LT-IIc toxins have one of three types of B polypeptides - one B subunit is present in theLT-IIc1 and LT-IIc5 toxins (14 isolates), and another is present in the LT-IIc3 subgroup (6 isolates) (Figure 6). A third B subunit is the most common variant and appears to be a recombinant between LT-IIc1/5 B and LT-IIc3 B. This B subunit, present in the LT-IIc2, -IIc4, and -IIc6 subgroups (28 isolates), has the signal sequence and first 22 residues of the LT-IIc1/IIc5 B subgroups (differing at nine residues, six of which are in the mature B polypeptide) while the remainder of the B subunit is identical to that of the LT-IIc3 subgroup members.

**Figure 7:**
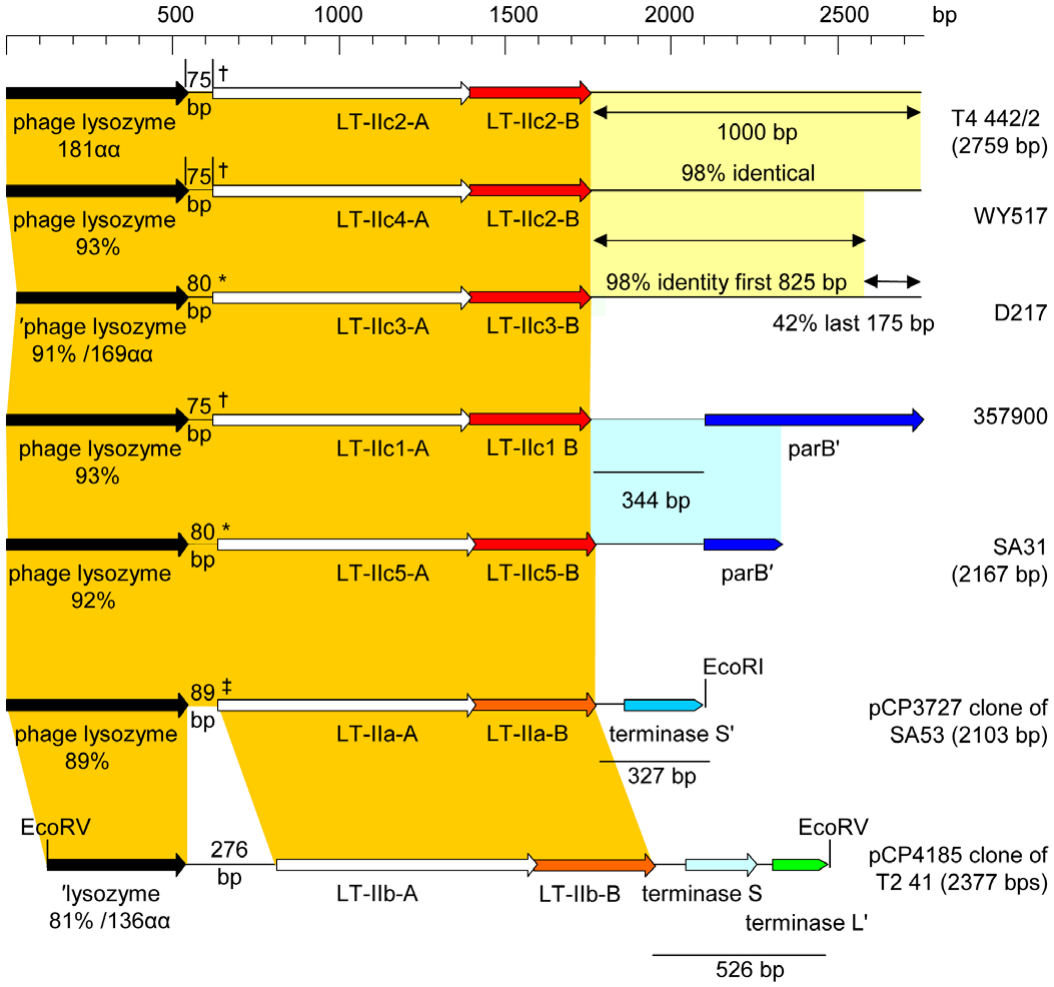
Graphical representation of gene organization of representative members of each LT-IIc subgroup, LT-IIa and LT-IIb. Scale bar in base pairs is shown at the top. Isolate or clone name is shown at the right. Homologous genes are like colored, and regions highly similar between loci are shaded like colors. The size of the lysozyme-A gene intergenic region is shown for each locus, with symbols denoting that: † DNA sequences are identical; * DNA sequences are identical, 69% identity to †; ‡ DNA sequence shows 81% identity to †, 67% identity to *. LT-IIc6 (not shown) has an identical arrangement to LT-IIc2 differing at only 6 bases in the first 1700 nucleotides of available sequence, save for the 14 additional bases of the A1 gene derived from the LT-IIc4 locus.

Additionally there are also minor variants within the LT-IIc5 B genes that also appear to have arisen by recombination between alleles. Two alleles (from SA76-4 and SA100 2nc) of the IIc5 subgroup have seven single nucleotide polymorphisms (SNPs), four of which are silent, in the B gene sequence that change three residues of the mature protein, G1D, T5N and A11G. The first six SNPs (and corresponding residues) are found in this region of the LT-IIc3 B genes (Figure S5). This suggests strongly that this region of the LT-IIc5 variants originated in an LT-IIc3 locus and recombined into an LT-IIc5 locus. Taken together, multiple recombination events appear to have taken place to form the six main subgroups of LT-IIc toxins. A complete DNA sequence comparison for representative members of these families is shown in Figure S5. Figure 6 shows the same comparison based on the A and B polypeptides, and Figure 7 gives a graphical representation of the domain combinations present in the toxins of these families.

The 6 subgroups of LT-IIc appear to have arisen from two ancestral toxins, IIc1 and IIc3, based on recombinational assortment of fragments of the operon (Figures 6 and S5). One scenario may be that toxins in subgroup IIc4 arose by recombination within a 9 bp segment of the B genes of IIc1 and IIc3, so that these toxins have the A gene and 5′ portion of the B gene of IIc1 and the remainder of the B gene of IIc3. Toxins in subgroups IIc2 and IIc6 appear to be IIc4-like with different segments of the A1 gene spliced in from IIc3 (Figure 6). They appear to have the same recombination event at the 5′ end to splice IIc3 and IIc1 A genes, followed by independent second recombination events within the A2 region of a IIc4 toxin. Since we do not have any examples of an intermediate toxin (IIc3 but with the amino-terminus of IIc1 A gene) another possibility is that a IIc4 toxin twice independently recombined with a IIc3 toxin to receive different fragments encoding the same carboxyl-terminal region of the A1 gene and varying portions of the A2 gene. Similarly, the LT-IIc5 subgroup could have arisen by recombination between LT-IIc3 and LT-IIc1 loci near the start of the B gene such that they have the A gene of IIc1 and the B gene of IIc3.

The chromosomal locations of LT-IIc operons suggest strain stability, but the evidence for multiple recombination events within the A and B genes demonstrated by these loci implies that different LT-II loci must sometimes be present, at least transiently, within the same cell. It is easy to see how this could occur if the loci are present on infectious phage, as can be seen in the case of Stx-converting lambdoid phages of EHEC, where co-infection and double lysogenic states have been documented [36]. That these LTII loci may be within lambdoid type prophages is suggested by the DNA sequences of the immediate flanking regions of the operons. Every LT-II operon, including LT-IIa and LT-IIb, has homologous genes 5′ of the A gene whose best BLAST matches are to predicted phage lysozymes (Figure 5), and while the 3′ sequences do not appear to be conserved across the LT-II family, at least in the immediate 1 kb of DNA, the best BLAST matches for each 3′ sequence are all phage-related. It would appear that the LT-II toxins may represent another example of phage conversion. Phage-borne virulence factor loci often appear to be morons – that is, genes located between two genes whose homologues are adjacent in related phage [37], [38], [39]. Whether this is the case for the LT-II loci remains to be determined.

The *stx* genes of EHEC temperate lambdoid-like phages certainly belong to this class of genes; their location in the phage is also similar, as the *stx* genes are present immediately 5′ of the lysis genes, whereas the LT-II loci are immediately 3′ of a lysis gene. This may also have implications for toxin production, since it has been shown that induction of the *stx* genes occurs upon induction of the lysogenic prophage, leading to upregulation of toxin expression [40], and this could potentially also occur with the LT-II loci.

We know very little about the prevalence and distribution of type II ETEC and their role in disease. Table 2 summarizes the properties of isolates organized by toxin subgroup, which shows little clear division by disease state (where known), geographical location or phylotype. Only IIc1variants show 100% association with animals with diarrhea, although the number of isolates is too small to be conclusive. Members of every major phylogenic class of *E. coli* (A, B1, D1, D2) are represented by these isolates, and they vary widely in serotype, when that has been determined. Nothing is known about any colonization factors that may be present in these isolates, and only a handful have been tested for the presence of other virulence factors. Of the 50 type II ETEC isolates presented here, the majority (30, 60%) came from ungulates – 29 bovines – cattle or buffalo, the majority of these being sick calves, and one sheep. Ten (20%) of the isolates came from humans, and a majority of them (60%) were isolated from patients with diarrhea. Four of the 6 LT-IIc subgroups (covering 88% of LT-II isolates) include an isolate that came from a human with diarrhea, and geographical origins of these isolates are global (Brazil, Bangladesh, Thailand and Africa). The remaining ten isolates came from food samples in Brazil or Thailand, and this set includes the sole LT-IIb isolate. The three LT-IIa isolates came from an asymptomatic human in the Philippines, a buffalo in Thailand and a sick cow in Sri Lanka. Our isolates were mostly collected in the 1980's, although some isolates from the National Animal Disease Center were collected much earlier. The lack of reports of more recent isolates is likely due to the lack of convenient tools to detect their presence. Our collection is derived from mainly two investigators – Trabulsi in Brazil and Echeverria in Thailand. The latter isolates designated SA came from an epidemiological study of diarrhea among rural Thai villagers and were collected from water buffalo kept under the home of an index case of diarrhea. A third group (C1–C9) is a subset of 20 isolates from Mainil in Belgium that were described as LT-II probe positive ETEC from sick cattle in a note published in 1987 [41]. The literature reports that both LT-IIa bovine isolates also tested positive for SLT-II (VT2) [15]. The single ovine isolate (septicemia) in this collection (obtained from the National Animal Disease Center) is the S5 type isolate for Vir+ *E. coli* [42], known to also produce the type 2 cytotoxic necrotizing factor (CNF2). Oswald characterized a broad collection of necrotoxic *E. coli* (NTEC) from animals with diarrhea or septicemia for the presence of CNF2 [43] and found several LT-IIa-probe positive isolates, but they were not characterized further. The isolate encoding the LT-IIc prototype is one of four from a South American outbreak of diarrhea in ostriches [17] and is the only avian LT-II-producing isolate known so far.

Literature searches identified a total of 76 isolates other than SA53 (LT-IIa), T2 41 (LT-IIb) or the four ostrich OS1-4 isolates (LT-IIc), annotated or detected as type II ETEC from a variety of sources, mostly South American animal or food isolates [41], [44], [45], [46], [47], [48], [49]. Two reports of approximately 50% carriage of LT-II-probe positive *E. coli* by pigs [50], [51] are probably false positives [52]. Pohl described 11 other isolates from Belgian calves in addition to the nine isolates included here [41]. Another eight LT-II producing isolates were identified in cattle in Sri Lanka [49], part of a longitudinal study of SLT-II-encoding EHEC (VTEC) carriage. One SLT-II isolate also produced LT-II, as did 7 of 30 isolates subsequently found to have lost SLT activity. Only three of these eight isolates were LT-IIa probe positive, while all were positive with a probe derived from the LT-II toxin locus from isolate 357900 [53], which we have here determined to encode LT-IIc1. These two probes have a 749 bp region in common that encodes parts of the A and B genes, 343 bp of which are 96% identical (A1 gene) and 406 bp of the A2 and B genes that shows only 59% identity. It is likely therefore that these too are LT-IIc isolates. Two studies [44], [45] on ETEC in Brazilian calves with diarrhea identified 49 isolates that were PCR positive using LT-IIa primers, nine of which in one study were also STa-positive; three of 17 isolates in the other study were also positive for CNF2, STa or both (one isolate each). Three other studies between them identified eight other LTII isolates [46], [47], [48] from milk, calf stool or ground beef samples, respectively.

In the US, PCR probes specific for LT-IIa have been used as a surrogate biomarker for cattle fecal pollution [54], [55], [56] and these studies detected LT-IIa DNA in up to 100% of lagoon waste water samples, suggesting that LT-IIa isolates may be frequently found in domestic cattle populations, with potential for contamination of the food supply. Since in our collection, LT-IIc isolates are much more common than LT-IIa isolates, and the majority of these came from cattle, the prevalence type II ETEC isolates could have been greatly underestimated by using primers or probes specific for LT-IIa only.

In summary, we characterized a novel set of type II heat-labile enterotoxins that constitute the LT-IIc group, and we showed that members of the LT-IIc group predominate in our strain collection. The previously known members of the LT-II family, LT-IIa and LT-IIb are represented by only four isolates, with three encoding LT-IIa and a single isolate encoding LT-IIb. Current evidence suggests that most uncharacterized type II ETEC isolates are likely to belong to the LT-IIc family. In every isolate where we characterized the LT-II locus by DNA sequence analysis, the LT-II encoding genes were flanked by homologous phage-related genes upstream and phage-related sequences downstream. This evidence suggests that the LT-II enterotoxins may be prophage-encoded. If LT-II loci are indeed present on functional phages, then they could probably disseminate widely. Type II ETEC isolates have been recovered from several humans with diarrhea, suggesting that they may be capable of causing human disease and may be responsible for more cases of diarrhea than currently recognized. A large number of our isolates were recovered from sick cattle, as were a majority of the other type II ETEC isolates in the literature, and the remaining isolates came from human foodstuffs. There is thus the likelihood of contamination of the food supply with organisms potentially capable of causing human disease, as has been seen with EHEC isolates [57]. How often this occurs is not known. The determination and analysis of the DNA sequences for a large collection of type II loci in ETEC, as reported here, provides important tools that will aid in answering these questions.

## Supporting Information

Figure S1 **Amino-acid comparison of the mature A polypeptides of LT, CT, LT-IIa and LT-IIb.** Residues are shown in lower case single-letter code for LT-I, and identical residues in the other proteins are shown as periods, residues that differ are shaded green. Bold case shows the regions chosen to design degenerate primers (open arrows); above is the primer sequence or the coding sequence of reverse primer; restriction sites are italicized, alternating codons are underlined).
(PDF)

Figure S2 **Novel DNA and protein sequence of 442/2 LT-II A1 gene compared to LT-IIa and LT-IIb.** The nucleotide (A) and deduced amino-acid (B) comparisons of the unique region of the PCR product amplified from strain 442/2 using degenerate primers are compared with the same regions of the LT-IIa and LT-IIb A genes and polypeptides.
(PDF)

Figure S3 **Nucleotide and deduced amino-acid sequences of the 442/2 LT-II locus.** DNA sequence of LT-IIc isolate 442/2 (1280 bp) compiled from walking and inverse PCR products is shown with translated open reading frames (A polypeptide above, B polypeptide below the DNA sequence), showing the positions and sequence of relevant PCR primers used (highlighted in yellow; lower case nucleotides in primers denote mismatches).
(PDF)

Figure S4 **Nucleotide sequence of LT-IIc (OS1) locus compared to LT-IIc (442/2).** A. DNA sequence for OS1 shown in lower case (with start and stop codons for the A and B genes shown in bold capitals). Periods show identical bases for the 442/2 isolate. Nucleotide differences are shown and shaded in light green.
(PDF)

Figure S5 **Amino-acid sequence comparisons between LT-IIc(OS1), LT-IIc(442/2) and LT-IIa and LT-IIb.** A polypeptides (upper panel) and B polypeptides (lower panel). Periods show identical residues to LT-IIc(OS1), differences are shown and shaded in light green. Signal sequences are underlined.
(PDF)

Figure S6 **Nucleotide sequence comparison of all LT-IIc subgroups**. DNA sequences are color coded to show domain homologies – IIc3-like in light green, IIc1 like in pink. Sequences are identified by subgroup and the strain name. IIc1 357900 is identical to IIc1 OS1. Potential recombination regions at domain boundaries are shown in yellow. Bases differing from consensus for domain (presumed mutations) are not colored. Sequences start with the ATG for the A genes through the stop codons (in bold capitals) for the B genes. Dashes indicate bases for IIc5var from SA100 (and SA76) have not been determined. Sequence is shown for the IIc1 variant group (NADC567 and 1034, 336A, 30401-3 and 30580-3) from base 631 through 1090 only in the region where they differ from the rest of IIc1.
(PDF)
