# Supplementary material for: Type II Heat-Labile Enterotoxins from 50 Diverse Escherichia coli Isolates Belong Almost Exclusively to the LT-IIc Family and May Be Prophage Encoded
Source: PLoS One. 2012 Jan 5;7(1):e29898. doi: 10.1371/journal.pone.0029898 (PMC3252337; doi:10.1371/journal.pone.0029898)
Supplement: Figure S1 — Amino-acid comparison of the mature A polypeptides of LT, CT, LT-IIa and LT-IIb. Residues are shown in lower case single-letter code for LT-I, and identical residues in the other proteins are shown as periods, residues that differ are shaded green. Bold case shows the regions chosen to design degenerate primers (open arrows); above is the primer sequence or the coding sequence of reverse primer; restriction sites are italicized, alternating codons are underlined). (PDF) [file pone.0029898.s001.pdf]

```
        5′ GAGCwGAyTCTAGAmCnCCwGAyGA 3′

hLT          ngdklyradsrppdeikrsgglmprghneyfdrgtqmninlydhargtqt
pLT          ...r..............................................
CT           .d...............q........qs......................
IIa          --ndff.....t....r.a...l...qq.aye...pi.....e.....v.
IIb          --ndyf.....t...vr.....i...qd.aye...pi...........a.

hLT          gfvryddgyvstslslrsahlagqsilsgystyyiyviatapnmfnvndv
pLT         ...................t..............................
CTA          ....h........i.......v..t....h....................
IIa          .nt..n......tvt..q...i..n..gs.ne.....v.p...l.d..g.
IIb          .nt..n......ttt..q..fl..nm.g..ne.....v.a...l.d..g.

hLT          lgvysphpyeqevsalggipysqiygwyrvnfgviderlhrnreyrdryy
pLT          ..................................................
CT           ..a.....d.....................h...l..q.....g......
IIa          ..r...y.s.n.fa......l...i.....s..a.eggmq...d..gdlf
IIb          ..r...y.s.n.ya......l...i.....s..a.eggm....d..rdlf


                          5′ GCnTGGrrrGAArsGCCATGGrC 3′

hLT          rnlniapaedgyrlagfppdhqawreepwihhapqgcgnssrtitgdtcn
pLT          ...........................................m......
CT           s..d....a...g......e.r............p....ap.ssmsn..d
IIa          .g.tv..n....q.....snfp....m..stf..eq.vpnnkefk.gv.i
IIb          .g.sa..n.....i....dgfp..e.v..ref..ns.lpnnkassdt..a

hLT          eetqnlstiylrkyqskvkrq-----i-fsdyqsevdiy--nrirnel*
pLT          ............e........-----.-...........--....d..*
CT           .k..s.gvkf.de........-----.-..g...di.th--...kd..*
IIa          sa.nv..kyd.mnfkkll..r-----laltffm..d.figvhge.d..*
IIb          sl.nk..qhd.adfkkyi..kftlmtl-l.inndgffsn--.ggkd..*
```
