# Supplementary material for: Type II Heat-Labile Enterotoxins from 50 Diverse Escherichia coli Isolates Belong Almost Exclusively to the LT-IIc Family and May Be Prophage Encoded
Source: PLoS One. 2012 Jan 5;7(1):e29898. doi: 10.1371/journal.pone.0029898 (PMC3252337; doi:10.1371/journal.pone.0029898)
Supplement: Figure S2 — Novel DNA and protein sequence of 442/2 LT-II A1 gene compared to LT-IIa and LT-IIb. The nucleotide (A) and deduced amino-acid (B) comparisons of the unique region of the PCR product amplified from strain 442/2 using degenerate primers are compared with the same regions of the LT-IIa and LT-IIb A genes and polypeptides. (PDF) [file pone.0029898.s002.pdf]

A

```
4422       1 gaaataagacgtgcgggagggcttttaccaagagggcagcaggaggcttatgagcgcgga
IIa        1 ............................................a...............
IIb        1 ...g.c.....ct.a..g...t.ga t........t...g.t...........a..t..c

4422      61 acaccaattaacatcaatctgtatgatcatgctcgcggaactgtaacggggaacaccaga
IIa       61 ..g.......................g..............a..................
IIb       61 ..g..t..a.....t..c..a.....c.....a..a..t....c...a........t...

4422     121 tataatgatgggtatgtatctactactactacgctgagacaggctcatttaatagggcag
IIa      121 ...........a...........a...gta...t..........................
IIb      121  ...........a........a..a..a..g..tt.............c..ct.......a

4422     181 aatctgtttggcagttataatgaatattacatatatgtagtcgcaccagcaccaaattta
IIa      181 ...a.ac.....................................................
IIb      181 ...a..c....tg.g........g..c..t..t.....t..t..tg.............g

4422     241 tttgatgtgaatggtgtgttaggacggtatagtccatatcccagtgaaaacgaatttgct
IIa      241 ..............a...................................t.........
IIb      241 ........a.....c..t.....ca..........g.....a........t....a....

4422     301 gcattaggtgggattcccttatcacaaattataggctggtatagagtatcttttggcgtg
IIa      301 ........a...............................................t.c.
IIb      301 ...........t......c.g........a..t....................c..t.ct

4422     361 atagaaggggaatgcagcgaaacaggcattatagaagagatttatttcaaggcttatcg
IIa      361 ...........................g.....c..g.............g...g...a..
IIb      361 .....g...........t.........g....c...............ag...g.....t

4422     361 gttgctcctaatcatgatggctatcatctcgcaggatttccagacggttttgccgc
IIa      361 .....a......g.a...........a..t.....g.....gagtaac..cc.a..
IIb      361 .c..........g.g..........gaa.t..t........c.....a...c.t..
```

B

```
4422 eirraggllprgqqeayergtpininlydhargtvtgntryndgyvsttttlrqahligq
IIa  ............................e....................v..........
IIb  .v..s...i....d....................a.....................fl..

4422 nlfgsyneyyiyvvapapnlfdvngvlgryspypsenefaalggiplsqiigwyrvsfgv
IIa  .il........................................................a
IIb  .ml.g..........a......................y....................a

4422 ieggmqrnrhyrrdlfqglsvapnhdgyhlagfpdgfaa
IIa  .........d..g...r..t....e...q.....sn.p.
IIb  .....h...d......r...a...e...ri.......p.
```
