# Supplementary material for: Type II Heat-Labile Enterotoxins from 50 Diverse Escherichia coli Isolates Belong Almost Exclusively to the LT-IIc Family and May Be Prophage Encoded
Source: PLoS One. 2012 Jan 5;7(1):e29898. doi: 10.1371/journal.pone.0029898 (PMC3252337; doi:10.1371/journal.pone.0029898)
Supplement: Figure S3 — Nucleotide and deduced amino-acid sequences of the 442/2 LT-II locus. DNA sequence of LT-IIc isolate 442/2 (1280 bp) compiled from walking and inverse PCR products is shown with translated open reading frames (A polypeptide above, B polypeptide below the DNA sequence), showing the positions and sequence of relevant PCR primers used (highlighted in yellow; lower case nucleotides in primers denote mismatches). (PDF) [file pone.0029898.s003.pdf]

```
   1  TATAGCTGGA TTTAGATAGA TTATAAAAAA TCACCTTTAA TAATATTTTT TAAATGGATA GGGTGTTGTG AAAAATTAAA
                                                                   IIaNdeF >
                                                       GcCca tatgAACGAT TTCTTTAGAG CAGAC
  81  ATGATTAAGC ATGTATTGTT GTTTTTTGTT TTTATATCAT TTTCTGTCTC AGCAAACGAT TTCTTTAGAG CAGACTCCAG
        m  i  k   h  v  l   l  f  f  v   f  i  s   f  s  v   s  a  n  d   f  f  r   a  d  s
                 < 44225XR
          GGTCTA CTTTATTCTG CACG
 161  AACACCAGAT GAAATAAGAC GTGCGGGAGG GCTTTTACCA AGAGGGCAGC AGGAGGCTTA TGAGCGCGGA ACACCAATTA
      r  t  p  d  e  i  r   r  a  g   g  l  l  p   r  g  q   q  e  a   y  e  r  g   t  p   i

 241  ACATCAATCT GTATGATCAT GCTCGCGGAA CTGTAACGGG GAACACCAGA TATAATGATG GGTATGTATC TACTACTACT
       n  i  n   l  y  d  h   a  r  g   t  v  t   g  n  t  r   y  n  d   g  y  v   s  t  t  t

 321  ACGCTGAGAC AGGCTCATTT AATAGGGCAG AATCTGCTTG GCAGTTATAA TGAATATTAC ATATATGTAG TCGCACCAGC
        t  l  r   q  a  h   l  i  g  q   n  l  l   g  s  y   n  e  y  y   i  y  v   v  a  p

 401  ACCAAATTTA TTTGATGTGA ATGGTGTGTT AGGACGGTAT AGTCCATATC CCAGTGAAAA CGAATTTGCT GCATTAGGTG
      a  p  n  l  f  d  v   n  g  v   l  g  r  y   s  p  y   p  s  e   n  e  f  a   a  l   g
                                         < 4422R2
                               CCGACCA TATCTCATAG AAAACCGC
 481  GGATTCCCTT ATCACAAATT ATAGGCTGGT ATAGAGTATC TTTTGGCGTG ATAGAAGGGG GAATGCAGCG AAACAGGCAT
       g  i  p   l  s  q  i   i  g  w   y  r  v   s  f  g  v   i  e  g   g  m  q   r  n  r  h
                                                                  4422NF >
                                                       GATGG CTATCATCTC GCAGGATTTC C
 561  TATAGAAGAG ATTTATTTCA AGGCTTATCG GTTGCTCCTA ATCATGATGG CTATCATCTC GCAGGATTTC CAGACGGTTT
        y  r  r   d  l  f   q  g  l  s   v  a  p   n  h  d   g  y  h  l   a  g  f   p  d  g

 641  TGCCGCATGG CGAGAGCTGC CGTGGAGTGC ATTTGCTCCG GAACAGTGTG AGCAAGATTA CATGGTTAGA AATTTAGATG
      f  a  a  w  r  e  l   p  w  s   a  f  a  p   e  q  c   e  q  d   y  m  v  r   n  l   d

 721  CCTGCGATTC TTATACAAAT ATATTATCTC AAAATGATTT GGTCGCTTTT AAAAGATTTA TGCGAATTCG TTCTTCCCTT
       a  c  d   s  y  t  n   i  l  s   q  n  d   l  v  a  f   k  r  f   m  r  i   r  s  s  l

 801  ATGATTTTAC AAAGTATTGA GGATGATTTA CAAGACAATG AAAATAAAGA TGAACTTTAA AAAGTCAATT GCGTTGTTGT
        m  i  l   q  s  i   e  d  d  l   q  d  n   e  n  k   d  e  l  -
                                                              m  n  f   k  k  s  i   a  l  l

 881  TTATTGCCTT AAATATTGCA TCACTACCAA CATATGCTGG CGTAAGTAAA ACTTTTAAGG ATAAATGCGC TTCTACTACG
       f  i  a   l  n  i  a   s  l  p   t  y  a   g  v  s  k   t  f  k   d  k  c   a  s  t  t
                     iixBF >
            CTTG TACAGAGTGT TCAGTTGG
 961  GCCAAACTTG TACAGAGTGT TCAGTTGGTA AAACTAGCAT CTGATACCAA CAAGGACAGT AAGGGTATTT ATATAACCGA
        a  k  l   v  q  s   v  q  l  v   k  l  a   s  d  t   n  k  d  s   k  g  i   y  i  t

1041  TTCTACAGGA AAAACCAGAT TCATTCCTGG GGGGCAGTAC TATCCCGAGA ATTATCTGAG CAATGAGATG AGGAAAATAG
      d  s  t  g  k  t  r   f  i  p   g  g  q  y   y  p  e   n  y  l   s  n  e  m   r  k   i
                                                                                 < IIxBecoRIR
                                                                                    ACCCGTTAA
1121  CAATGGCTGC GGTGCTTTCT AATGTTAGGG TAAATATCTG TGCGAGCGAA GCATATACTC CTAATCACGT ATGGGCAATT
       a  m  a   a  v  l  s   n  v  r   v  n  i   c  a  s  e   a  y  t   p  n  h   v  w  a  i

      CTTAATCGTg GtCccTtaag gcg
1201  GAATTAGCAG CGGAATAGCT AATTGTAGTT TTTATTGGGG TTTGTTGAGT ATTAGAAGGT GTTAAATTGT ATGAATGTGT
        e  l  a   a  e  -
```
