# Supplementary material for: Type II Heat-Labile Enterotoxins from 50 Diverse Escherichia coli Isolates Belong Almost Exclusively to the LT-IIc Family and May Be Prophage Encoded
Source: PLoS One. 2012 Jan 5;7(1):e29898. doi: 10.1371/journal.pone.0029898 (PMC3252337; doi:10.1371/journal.pone.0029898)
Supplement: Figure S4 — Nucleotide sequence of LT-IIc (OS1) locus compared to LT-IIc (442/2). A. DNA sequence for OS1 shown in lower case (with start and stop codons for the A and B genes shown in bold capitals). Periods show identical bases for the 442/2 isolate. Nucleotide differences are shown and shaded in light green. (PDF) [file pone.0029898.s004.pdf]

OS1	1	**ATG**attaagcatgtattgttgtttttttgttttttatatcattttctgtctcagcaaacgatttctttagagcagactccag
4422	1	....
OS1	81	aacaccagatgaaataagacgtgcgggagggcttttaccaagagggcagcaggaggcttatgagcgcggaactccaatta
4422	81	....a.......
OS1	161	acatcaatctgtatgagcatgctcgcggaacagtaacggggaacactagatataacgatgggtatgtatctacaactaca
4422	161	................t..............t..............c........t.................t.....t
OS1	241	actttgagacaggctcatttaatcgggcagaatatacttggcagttataatgaatattacatatatgtagttgcaccagc
4422	241	..gc...................a.........c.g...................................c........
OS1	321	acccaatttatttgatgtgaatggtgtgttagggcggtatagtccatatcccagtgaaaatgaatttgctgcattaggag
4422	321	...a.............................a..........................c.................t.
OS1	401	ggattcccttatcacaaattataggctggtatagagtatcttttggtgcgatagaaggggaatgcagcgaaacaggcat
4422	401	....c.t...............................
OS1	481	tacagaggagatttatttcaaggcttatcggttgcccctaatcatgatggctatcatctcgcaggatttccagagggttt
4422	481	..t...a............................t......................................c.....
OS1	561	tgctgcatggcgagagctgccatggagtgcatttgctccggaacagtgcgagcaagattacatggttagaaatttagatg
4422	561	...c.................g..........................t...............................
OS1	641	cctgcgattcttatacaaatatattatctcaaaatgatttggtcgcttttaaaagatttatgcgaattcgttcttccctt
4422	641	....
OS1	721	atgattttacaaagtattgaggatgatttacaaaacaatgaaaataaag**ATG**aactt**TAA**aaagtcaattgcgttgttgt
4422	721	.................................g..
OS1	801	ttattgccttaaatattgcatcactaccaacatatgctggcgtaagtaaaacttttaaggataaatgcgcttcgactacg
4422	801	....t......
OS1	881	gccaagcttgtacagagtgttcagttggtaaatatctcatctgatgtaaataaggacagcaagggaatttatatatcaag
4422	881	.....a..........................ac.ag........acc..c........t.....t.........a.cga
OS1	961	ctcagcaggaaaaacatggtttattccgggggggcagtattaccctgataactatctaagtaatgaaatgagaaaaatag
4422	961	t..ta..........ca.a..c.....t...........c..t..c..g..t.....g..c.....g.....g.......
OS1	1041	caatggctgcagttctttctaacgtaagggtaaatctatgtgcgagtgaagcatatactccgaatcatgtatgggcaatt
4422	1041	..........g..g........t..t.........a.c........c..............t.....c............
OS1	1121	gaattagcacca**TAA**tag
4422	1121	.........g.gg..**TAG**
