# Supplementary material for: Type II Heat-Labile Enterotoxins from 50 Diverse Escherichia coli Isolates Belong Almost Exclusively to the LT-IIc Family and May Be Prophage Encoded
Source: PLoS One. 2012 Jan 5;7(1):e29898. doi: 10.1371/journal.pone.0029898 (PMC3252337; doi:10.1371/journal.pone.0029898)
Supplement: Figure S5 — Amino-acid sequence comparisons between LT-IIc(OS1), LT-IIc(442/2) and LT-IIa and LT-IIb. A polypeptides (upper panel) and B polypeptides (lower panel). Periods show identical residues to LT-IIc(OS1), differences are shown and shaded in light green. Signal sequences are underlined. (PDF) [file pone.0029898.s005.pdf]

```
IIc1 A OS1     1 mikhvllffv---fisfsvsandffradsrtpdeirraggllprgqqeayergtpininlyehargtvtgntryndgyvsttttlrqahl
IIc2 A 442/2   1 ..........---................................................d............................
IIa A          1 ..........---.....................................................................v.......
IIb A          1 .-ak.is..islfl...ply...y..........v..s...i....d..............d.....a.....................f

IIc1 A OS1    88 igqnilgsyneyyiyvvapapnlfdvngvlgryspypsenefaalggiplsqiigwyrvsfgaieggmqrnrhyrgdlfqglsvapnhdg
IIc2 A 442/2  88 ....l.........................................................v............r..............
IIa A         88 ........................................................................d......r..t....e..
IIb A         90 l...m..g..........a......................y..........................h...d..r...r...a...e..

IIc1 A OS1   178 yhlagfpegfaawrelpwsafapeqceqdymvrnldacdsytnilsqndlvafkrfmrirsslmilqsiedd--lqnnenkdel
IIc2 A 442/2 178 .......d................................................................--..d.......
IIa A        178 .q.....sn.p....m...t......vpnnkefkggv.i.a..v..ky..mn..kllkr.la.tffm.ed.f--igvhger...
IIb A        180 .ri....d..p..e.v..re...ns.lpnnkassdtt.a.l..k...h..ad..kyikrkft..t.l..nn.gffs..gg....

IIc1 B         1 mnfkksiallfialniaslptyagvsktfkdkcasttaklvqsvqlvnissdvnkdskgiyisssagktw
IIc2 B         1 ...............................................kla..t.........td.t...r
IIa B          1 .ss..i.gafvlmtg.l.gqv.....eh.rni.nq...di.ag...kkyia...tntr...vv.nt.gv.
IIb B          1 .s...i.kafv.maalv.vqah..a.qf...n.nr...s..eg.e.tkyi..i.nntd.m.vv..t.gv.

IIc1 B        61 fipggqyypdnylsnemrkiamaavlsnvrvnlcaseaytpnhvwaielap**-
IIc2 B        61 .........e......................i.................ae*-
IIa B         61 y....rd....f..g.i..t....i..dtk.....ktsss...i..m..dres*
IIb B         61 r.srakd....vmta............gm...m...p.ss..vi.....eae*-
```
