# Supplementary material for: Type II Heat-Labile Enterotoxins from 50 Diverse Escherichia coli Isolates Belong Almost Exclusively to the LT-IIc Family and May Be Prophage Encoded
Source: PLoS One. 2012 Jan 5;7(1):e29898. doi: 10.1371/journal.pone.0029898 (PMC3252337; doi:10.1371/journal.pone.0029898)
Supplement: Figure S6 — Nucleotide sequence comparison of all LT-IIc subgroups. DNA sequences are color coded to show domain homologies – IIc3-like in light green, IIc1 like in pink. Sequences are identified by subgroup and the strain name. IIc1 357900 is identical to IIc1 OS1. Potential recombination regions at domain boundaries are shown in yellow. Bases differing from consensus for domain (presumed mutations) are not colored. Sequences start with the ATG for the A genes through the stop codons (in bold capitals) for the B genes. Dashes indicate bases for IIc5var from SA100 (and SA76) have not been determined. Sequence is shown for the IIc1 variant group (NADC567 and 1034, 336A, 30401-3 and 30580-3) from base 631 through 1090 only in the region where they differ from the rest of IIc1. (PDF) [file pone.0029898.s006.pdf]

```
IIc3 D217         1 ATGattaaacatttattgttgttttttgtttttatatcattttctgtctcagcaaatgatttttttagagcagacaccagaacacctagt
IIc1 357900       1 ........g...g...........................................c.....c............t..........aga.
IIc2 442/2        1 ........g...g...........................................c.....c............t..........aga.
IIc6 C1           1 ........g...g...........................................c.....c............t..........aga.
IIc4 WY517        1 ........g...g...........................................c.....c............t..........aga.
IIc5 var SA100    1 ------------------------------------------------------------------------------............
IIc5 SA31         1 ..........................................................................................

IIc3 D217        91 gaaataagacaagctggaggacttttgcctcgaggtcagcaagaggcttatgagcgcggaacaccaattaacatcaatctgtatgatcat
IIc1 357900      91 ..........gt..g.....g.....a..aa....g.....g....................t.......................g...
IIc2 442/2       91 ..........gt..g.....g.....a..aa....g.....g................................................
IIc6 C1          91 ..........gt..g.....g.....a..aa....g.....g................................................
IIc4 WY517       91 ..........gt..g.....g.....a..aa....g.....g....................t.......................g...
IIc5 var SA100   91 ..........................................................................................
IIc5 SA31        91 ..........................................................................................

IIc3 D217       181 gctcgcggaactgtaacggggaacaccagatataatgatgggtatgtatctactactactacgctgagacaggctcatttaatagggcag
IIc1 357900     181 ...........a..............t........c.................a.....a..tt...................c......
IIc2 442/2      181 ..........................................................................................
IIc6 C1         181 ..........................................................................................
IIc4 WY517      181 ...........a..............t........c.................a.....a..tt...................c......
IIc5 var SA100  181 ..........................................................................................
IIc5 SA31       181 ..........................................................................................

IIc3 D217       271 aatctgcttggcagttataatgaatattacatatatgtagtcgcaccagcaccaaatttatttgatgtgaatggtgtgttaggacggtat
IIc1 357900     271 ...a.a...................................t...........c.............................g......
IIc2 442/2      271 ..........................................................................................
IIc6 C1         271 ..........................................................................................
IIc4 WY517      271 ...a.a...................................t...........c.............................g......
IIc5 var SA100  271 ..........................................................................................
IIc5 SA31       271 ..........................................................................................

IIc3 D217       361 agtccatatcccagtgaaaacgaatttgctgcattaggtgggattcccttatcacaaattataggctggtatagagtatcttttggcgtg
IIc1 357900     361 ....................t.................a...............................................t.c.
IIc2 442/2      361 ..........................................................................................
IIc6 C1         361 ..........................................................................................
IIc4 WY517      361 ....................t.................a...............................................t.c.
IIc5 var SA100  361 ..........................................................................................
IIc5 SA31       361 ..........................................................................................

IIc3 D217       451 atagaagggggaatgcagcgaaacaggcattatagaagagatttatttcaaggcttatcggttgctcctaatcatgatggctatcatctc
IIc1 357900     451 ................................c...g............................c........................
IIc2 442/2      451 ..........................................................................................
IIc6 C1         451 ..........................................................................................
IIc4 WY517      451 ................................c...g............................c........................
IIc5 var SA100  451 ..........................................................................................
IIc5 SA31       451 ..........................................................................................

IIc3 D217       541 gcaggatttccagacggttttgccgcatggcgagagctgccgtggagtgcatttgctcctgctgcatgcgaacatgattacatggttcga
IIc1 357900     541 ..............g........t.................a.................g.aacag.....g..a............a..
IIc2 442/2      541 ...........................................................g.aacag..t..g..a............a..
IIc6 C1         541 ..........................................................................................
IIc4 WY517      541 ..............g........t.................a.................g.aaca......g..a............a..
IIc5 var SA100  541 ..........................................................................................
IIc5 SA31       541 ..........................................................................................

IIc3 D217       631 attttagatgcctgcgattcttatacgaatagaatatctaaaaatgatttatttgcttttaaaagatttatgcggattcgctcttctctg
IIc1 357900     631 .a........................a....t.t.....c..........gg.c....................a.....t.....c..t
IIc1 var        631 .a........................a....t.t.....c..........gg.c....................a.....t.....t..t
IIc2 442/2      631 .a........................a....t.t.....c..........gg.c....................a.....t.....c..t
IIc6 C1         631 .......................................c..........gg.c....................a.....t.....c..t
IIc4 WY517      631 .a........................a....t.t.....c..........gg.c....................a.....t.....c..t
IIc5 var SA100  631 ..................................................g.......................................
IIc5 SA31       631 ..................................................g.......................................

IIc3 D217       721 atgatcttacaaagtatcgaggatgatttacaatataatgaaaataaaaATGaacttTAAaaagtcaattgcattattgtttgttgtctt
IIc1 357900     721 .....t...........t...............a.c............g.......................g..g......a...c...
IIc1 var        721 .....t...........t...............a.c............g.......................g..g......a...c...
IIc2 442/2      721 .....t...........t...............g.c............g.......................g..g......a...c...
IIc6 C1         721 .....t...........t...............g.c............g.......................g..g......a...c...
IIc4 WY517      721 .....t...........t...............a.c............g.......................g..g......a...c...
IIc5 var SA100  721 ................................................g................t......g.........a.......
IIc5 SA31       721 ................................................g................t......g.........a.......
```

```
IIc3 D217        811 aaatattacatctctaccaacatatgctgatgtaagtaagaattttaaggataattgtggttctactacggccaaaattgtacaaagtgt
IIc1 357900      811 .......g....a................gc........a.c............a..c.c...g...........gc.......g.....
IIc1 var         811 .......g....a................gc........a.c............a..c.c...g........a..ac.......g.....
IIc2 442/2       811 .......g....a................gc........a.c............a..c.c................c.......g.....
IIc6 C1          811 .......g....a................gc........a.c............a..c.c................c.......g.....
IIc4 WY517       811 .......g....a................gc........a.c............a..c.c................c.......g.....
IIc5 var SA100   811 .......g....a...........................................................a...c.......g.....
IIc5 SA31        811 .......g....a................g.........a.c............c..c.c............a...c.......g.....

IIc3 D217        901 tcggttggtaaaactagcatctgataccaacaaggacagcaaaggtatttatataaccgattctacaggaaaaaccagattcattcctgg
IIc1 357900      901 ..a.........ta.ct........gta..t...........g..a.........t.aagc..ag..........at.g..t.....g..
IIc1 var         901 ..a.........ta.ct.g......gta..t........t..g..a.........t.aagc..ag..........at.g..t.....g..
IIc2 442/2       901 ..a....................................t..g...............................................
IIc6 C1          901 ..a....................................t..g...............................................
IIc4 WY517       901 ..a....................................t..g...............................................
IIc5 var SA100   901 ..a.........ta.ct.t......gta..t........t..g..a.........t.aagc..ag..........at.g..t.....g..
IIc5 SA31        901 ..a.........ta.ct.g......gta..t........t..g..a.........t.aagc..ag..........at.g..t.....g..

IIc3 D217       1091 ggggcagtactatcccgagaattatctgagcaatgagatgaggaaaatagcaatggctgcggtgctttctaatgttagggtaaatatctg
IIc1 357900     1091 .........t..c..t..t..c.....a..t.....a.....a.................a..t........c..a.........c.a..
IIc2 442/2      1091 ..........................................................................................
IIc6 C1         1091 ..........................................................................................
IIc4 WY517      1091 ..........................................................................................
IIc5 var SA100  1091 .........t..c..t..t..c.....a..t.....a.....a.................a..t........c..a.........c.a..
IIc5 SA31       1091 .........t..c..t..t..c.....a..t.....a.....a.................a..t........c..a.........c.a..

IIc3 D217       1181 tgcgagcgaagcatatactcctaatcacgtatgggcaattgaattagcagcggaaTAG
IIc1 357900     1181 ......t..............g.....t.....................c.aT.....
IIc2 442/2      1181 ..........................................................
IIc6 C1         1181 ..........................................................
IIc4 WY517      1181 ..........................................................
IIc5 var SA100  1181 ......t..............g.....t.................g...c.aT.....
IIc5 SA31       1181 ......t..............g.....t.................g...c.aT.....
```
